# Detection of Beta-Lactamases (ESBL and MBL) Producing Gram-Negative Pathogens in National Public Health Laboratory of Nepal

**DOI:** 10.1155/2022/5474388

**Published:** 2022-10-06

**Authors:** Anjana Shrestha, Jyoti Acharya, Jyoti Amatya, Rabin Paudyal, Nisha Rijal

**Affiliations:** ^1^Microbiology Department, National Public Health Laboratory, Teku, Kathmandu, Nepal; ^2^Department of Microbiology, Kathmandu College of Science and Technology, Kamalpokhari, Kathmandu, Nepal

## Abstract

**Background:**

Bacterial resistance to antibiotics has increased in recent years. Resistance to *β*-lactams in Gram-negative bacteria has been reported to be associated with extended spectrum beta-lactamases and metallo-beta-lactamases. This study was aimed at determining the distribution and antibiotic susceptibility patterns of Gram-negative pathogens producing extended spectrum beta lactamases and metallo-beta lactamases. *Method and Methodology*. This cross-sectional study was conducted at the National Public Health Laboratory during a period of six months. All clinical specimens were obtained and processed for the identification of Gram-negative pathogens by culture, morphological, and biochemical tests. Antibiotic susceptibility testing of the isolates was performed by the Kirby Bauer disc diffusion and the isolates were tested for ESBL and MBL by the combined disk method.

**Results:**

Out of 4266 clinical specimens, 197 (4.6%) were found to be Gram-negative bacterial isolates. 47 (23.9%) isolates were ESBL producers. The most predominant organisms were *Escherichia coli* (53%), *Klebsiella pneumonia* (23%), and *Pseudomonas* spp. (13%). 16 (8.2%) were positive for MBL producers, and 6(3.1%) were both ESBL and MBL producers. The MBL activity was seen in *E. coli* (38%), followed by *Pseudomonas* spp. (31%), and *K. pneumoniae* (19%). The ESBL producers showed a higher degree of sensitivity towards imipenem and amikacin, followed by piperacillin tazobactam. MBL producers showed sensitivity towards amikacin only.

**Conclusion:**

The prevalence of ESBL and MBL producing Gram-negative bacteria was found to be high in bacterial infections in Nepal. Routine laboratory testing for ESBL and MBL is needed in order to optimize antibiotic management and reduce the risk of spread of infections caused by ESBL and MBL producers.

## 1. Background

Gram-negative bacilli can cause serious infections in humans, both in community and hospital settings. Antibiotic resistance among Gram-negative bacilli is a rapidly increasing problem due to the organisms' ability to mutate and to acquire and transmit plasmids and other mobile genetic elements encoding resistance genes [1]. Multidrug resistance is a major health problem in Nepal that prevents the management of several infectious diseases and compromises therapy [2].

Beta-lactamase antibiotics are the most widely prescribed antibiotics worldwide, and the emergence of resistance to these agents has resulted in a major clinical crisis [3]. There are over 340 different types of *β*-lactamases. These are mainly ESBLs, AmpC, and carbapenems. ESBLs are still considered a threat since they are coded by plasmid and can be easily transmitted between species Carbapenems are used as the drug of choice to treat infections caused by ESBL-producing bacteria. However, over the past few years, carbapenem resistance due to metallo-beta-lactamases (MBLs) production has been increasingly reported among clinical isolates from all around the world [4].

MBLs have been globally isolated from various bacteria and more than 80 types of MBLs have been identified worldwide, with over 75% occurring as plasmid-encoded enzymes [5]. The rapid increasing rate of MBL production among the members of the Enterobacteriaceae, mainly *E. coli* and *K. pneumoniae*, which are the most common causes of infections among humans, is present as a serious global public health problem [4].

There are limited treatment options for infections caused by ESBL and MBL producing bacteria [6] because the treatments for such infections are very difficult, often resulting in treatment failure. The aim of the current study is to determine the prevalence of ESBL and MBL producers among Gram-negative clinical isolates. Early detection of ESBL and MBL producing organisms is crucial to establishing appropriate antimicrobial therapy and preventing their interhospital dissemination.

## 2. Materials and Methods

This cross-sectional study was conducted at the Bacteriology Department of National Public Health Laboratory (NPHL), Teku, during the period from October 2017 to March 2018. During the period, a total of 4266 different samples, including 3885 urine, 192 sputum, 44 pus, 70 body fluids, 72 throat swabs, 1 bile, and 2 tracheal aspirate samples from patients, were collected and processed. The growth of Gram-negative bacteria from the cultured samples was included in the study and was further tested for ESBL and MBL production. The ethical approval was obtained from the Ethical Review Board of the Nepal Health Research Council (NHRC) (Reg. 1289/2017).

### 2.1. Culture of Specimens

Culture of urine samples was done by a semiquantitative method on CLED (Cystine Lactose Electrolyte Deficient with Andrade indicator) agar plates. An inoculating loop of standard dimension was used to take up approximately fixed and a known volume (0.001 ml) of urine for inoculation. The urine specimens were thoroughly mixed to ensure uniform suspension of bacteria before inoculating the agar plates. If the culture indicated the presence of two uropathogens, both showing significant growth, definitive identification and antibiotic susceptibility testing of both were performed, whereas in cases of three pathogens, it was reported as mixed growth and asked for appropriate recollection with timely delivery to the laboratory (Cheesbrough [7]).

Other specimens like sputum, pus, body fluid, blood, and other cultures were inoculated into BA and MA plates and incubated at 35 ± 1°C for overnight in an aerobic condition.

### 2.2. Identification of the Isolates

The isolated colony showing significant growth from plates was further identified by using standard microbiological techniques which involved morphological appearance of the colonies, Gram's staining reactions, catalase test, oxidase test, and other biochemical tests. The biochemical media employed were Triple Sugar Iron agar (TSI), MR-VP, Sulphide Indole Motility (SIM) media, Simmons' citrate media, and Christensen's urease media.

### 2.3. Pure Culture for Identification

Each of the organisms was isolated in pure form before performing biochemical tests and antibiotic susceptibility tests. The single distinct colony was Gram stained and inoculated on the NA plate by using a sterile straight loop. Then the plate was incubated at 35 ± 1°C for 18–24 hours.

### 2.4. Antibiotic Susceptibility Test

Antibiotic susceptibility testing was performed on the different clinical isolates by the Kirby Bauer disc diffusion method using Mueller–Hinton Agar (MHA) following the guidelines of the Clinical and Laboratory Standards Institute [8].

### 2.5. Screening of ESBL Producing Strains

The initial screening test for the production of ESBL was performed by using both ceftazidime (CAZ) (30 *μ*g) and cefotaxime (CTX) (30 *μ*g) discs. If the zone of inhibition was ≤22 mm for CAZ and/or ≤27 mm for CTX, the isolate was considered as a potential ESBL producer as recommended by the Clinical and Laboratory Standards Institute [8].

### 2.6. Phenotypic Confirmatory Disc Diffusion Test for ESBL

Isolates that were suspected as ESBL producers by screening tests were tested further by the combined disc method for the confirmation of ESBL-producing strains in which a lawn culture of the isolated bacteria on Mueller–Hinton agar was made and CTX and CAZ (30 *μ*g), alone and in combination with clavulanic acid (CA) (10 *μ*g). A ≥ 5 mm increase in zone of inhibition for either antimicrobial agent tested in combination with CA versus its zone when tested alone confirmed ESBL, as per recommendations of CLSI [8].

### 2.7. Screening of MBL Producing Strains

The initial screening test for the production of MBL was performed by using ceftazidime (CAZ) and imipenem (10 *μ*g). If the zone of inhibition was ≤18 mm for CAZ and/or ≤19 mm for IPM, the isolate was considered as a potential MBL producer as recommended by the Clinical and Laboratory Standards Institute (CLSI) [8].

### 2.8. Phenotypic Confirmatory Disc Diffusion Test for MBL

0.5 M anhydrous Ethylene diamine-tetra acetic acid (EDTA) solution was prepared in distilled water. Its pH was adjusted to 8.0 using NaOH. The mixture was sterilized by autoclaving. Two imipenem or meropenem (10 *μ*g) discs were placed 25 mm (center to center) apart on the inoculated plate. One imipenem disc was enriched with EDTA by pouring 4 *μ*l of 0.5 M (750 *μ*g) EDTA onto it. After 16–18 hrs of incubation at 35 ± 1°C, the zone of inhibition around the imipenem disc was compared with the zone of inhibition around the EDTA-enriched disc. An increase in the zone diameter of >4 mm around the imipenem-EDTA disc compared to the imipenem disc alone was recorded as an MBL-positive [9].

### 2.9. Quality Control

The quality of each agar plate prepared was maintained by incubating one plate from each batch in the incubator. Control strains of ATCC were used for the identification test, for the standardization of the Kirby-Bauer test, and also for correct interpretation of the diameters of inhibition zones. The quality of the sensitivity test was maintained by maintaining the thickness of MHA at 4 mm and the pH at 7.2–7.4. Similarly, antibiotic discs containing the correct amount as indicated were used. Strict aseptic conditions were maintained while carrying out all the procedures.

The performance of newly prepared media was tested using control species of bacteria (i.e., known organisms giving positive and negative reactions). For stains and reagents, whenever new batches of them were prepared, a control smear was stained to ensure correct staining reaction. Control strains of *E. coli* (ATCC 25922) and *S. aureus* (ATCC 25923) were used for the quality control of the antibiotic sensitivity testing and for ESBL test standardization. *E. coli* ATCC 25922 and *K. pneumoniae* ATCC 700603 were used as negative and positive controls, respectively. For MBL test standardization, *P. aeruginosa* ATCC 27853 and *P. aeruginosa* PA 105663 were used as negative and positive controls, respectively.

The laboratory equipment was regularly monitored for its efficiency. The temperature of the refrigerator and incubator was monitored and documented every day.

### 2.10. Disposal of Sample and Used Media

All used samples were first dipped in hypochlorite solution for 30 minutes or overnight and then autoclaved. All media-containing Petri dishes and tubes were autoclaved and disposed.

### 2.11. Data Analysis

All the data collected were analyzed using MS Excel and Statistical Software SPSS version 16.0. Results were considered significant if *P* value was less than 0.05.

## 3. Results

### 3.1. Growth Profile in Clinical Samples

In this study, all together 4266 samples were processed, of which 3885 were urine, pus (44), sputum (192), body fluid (70), throat swab (72), tracheal aspirate (2), and one was a bile specimen. Among these, 197 (4.6%) showed the growth of Gram-negative isolates, while 4069 (93.4%) were culture negative for Gram-negative bacteria.

### 3.2. Distribution of Organisms among Different Clinical Samples

Out of 197 bacterial isolates, nine different bacteria were isolated. *Escherichia coli* (46.7%) was found to be the most predominant organism, followed by *Klebsiella* spp. (25.4%), *Pseudomonas* spp. (13.7%), *Citrobacter* spp. (6.6%), and *Proteus* spp. (4.06%), as shown in Table 1. Among the different samples, the highest growth was found to be from urine (78.7%), followed by sputum (15.2%). The growth of organisms among different clinical samples was found to be significant statistically (*P* value < 0.001).

### 3.3. ESBL Production among Gram-Negative Isolates

**Figure 1:**
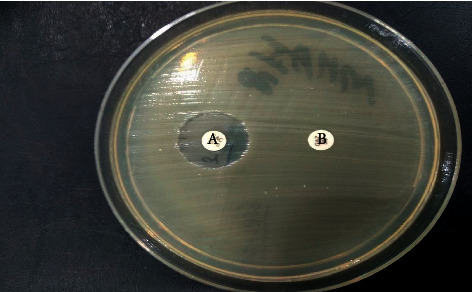
Confirmation of ESBL production by combined disc test in *Escherichia coli.* A = ceftazidime (30 mcg) with clavulanic acid (10 mcg) and B = ceftazidime (30 mcg), in this study.

88 were suspected of ESBL production among Gram-negative isolates. Among them, 47 (23.9%) were confirmed as ESBL producers by the combined disc method, as shown in Figure 1.

In the present study, the maximum ESBL activity was seen in *E. coli* (53%), followed by *K. pneumoniae* (23%). No ESBL activity was seen in *Klebsiella oxytoca*, *Proteus mirabilis*, *Burkholderia cepacia, Enterobacter aerogenes*, or *Citrobacter freundii* (Table 2).

### 3.4. MBL Production among Gram Negative Isolates

**Figure 2:**
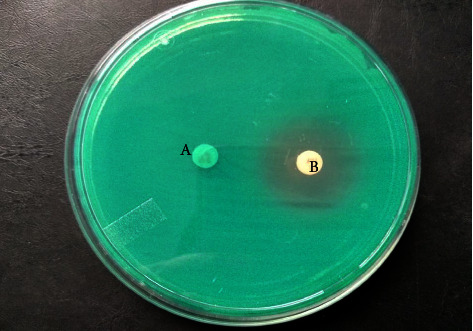
Confirmation of MBL production by combined disc diffusion method in *Pseudomonas* spp. (A = imipenem and B = imipenem + EDTA).

Among 28 suspected MBL producers, 16 (8.2%) were confirmed as MBL producers by the imipenem EDTA disc synergy method as shown in Figure 2. In the present study, the maximum MBL activity was seen in *E. coli* (38%). No ESBL activity was seen in *Klebsiella oxytoca, Citrobacter freundii*, or *Morganella morganii* (Table 3).

### 3.5. Antibiotic Resistance Pattern of ESBL Producers

All 47 ESBL positive isolates were subjected to primary and supplementary drugs for antibiotic susceptibility tests. It showed high resistance to being followed by cefotaxime (100%), ceftazidime (91.5%), ciprofloxacin (85.1%), and cotrimoxazole (83.0%). Most ESBL producers showed higher sensitivity towards imipenem (78.7%) and amikacin (73.3%), followed by piperacillin tazobactam (68.1%) (Table 4).

### 3.6. Antibiotic Resistance Pattern of MBL Producers

Of 16 MBL isolates, most showed high resistance towards cefepime (80%), piperacillin and tazobactam (75%), gentamycin (75%), and cefoperazone sulbactam (68%) and were found to be sensitive towards amikacin (44%), followed by meropenem (33%), and cefoperazone sulbactam (32%) (Table 5).

### 3.7. Coexistence of ESBL and MBL Producers among the Various Isolates

Among 197 Gram-negative isolates, 47(23.9%) were ESBL producers, 16 (8.2%) were MBL producers, and 6(3.1%) were both ESBL and MBL producers. The highest ESBL and MBL production was seen in *Escherichia coli* (50%), followed by *Klebsiella pneumoniae* (16.7%), *Pseudomonas* spp. (16.7%), and *Proteus vulgaris* (16.7%) (Table 6). The result was statistically significant (*P* value < 0.001).

## 4. Discussion

A total of 4266 clinical specimens (urine, body fluid, pus, bile, tracheal aspirate, throat swab, and sputum) were received for routine culture and susceptibility testing. Among them, 197 (4.6%) showed culture positive results and *E. coli* 92 (46.7%) was found to be the most predominant organism, followed by *Klebsiella* spp. 50 (25.4%), *Pseudomonas* spp. 2 (13.7%)*, Citrobacter* spp. 13 (6.6%), *Proteus* spp. 8 (4.06%), *Acinetobacte*r spp. 3 (1.5%), *Morganella* spp. 2 (1.01%), *Enterobacter* spp. 1 (0.5%), and *Burkholderia* spp. 1 (0.5%), respectively.

In this study, urine (78.7%) was the most prevalent specimen among six different clinical samples. *E. coli* (58.1%) was the most predominant isolate from urine, and *Pseudomonas* spp. (40%) was the predominant isolate from sputum, whereas *Klebsiella* (62.5%) was the predominant isolate from pus. *Acinetobacter* was isolated from tracheal aspirate, throat swab, and urine.

Extended spectrum beta-lactamase (ESBL) producing organisms create a major problem for clinical therapeutics. 47 (23.9%) were ESBL producers. Similar studies were conducted by Raut et al. [22] (22.4%), Nepal et al. (34.5%), and Pokharel et al. [21]. (16.0%). The major ESBL producers are *E. coli* (53%), followed by *K. pneumoniae* (23%)*, Pseudomonas* spp. (6%), and *Morganella morganii* (4%). The prevalence of ESBL-producing *E. coli* varies from country to country and from center to center. In this study, *E. coli* (53.33%) was the major ESBL producer. A similar study in Nepal on uropathogens showed that among the Gram-negative isolates, ESBL prevalence was highest in *E. coli*, followed by *K. pneumoniae* [10]. In Asia, the percentage of ESBL production in *E. coli* is 4.8, 8.5, and up to 12% in Korea, Taiwan, and Hong Kong, respectively [11–13]. In India, the percentage of ESBL producing *E. coli* ranges from 22 to 75% [23]. In Japan, the prevalence of ESBL-producing *E. coli* is <0.1% [14]. In the United States, ESBL producing *E. coli* ranges from 0 to 25%, with the average being around 3% [15].

In this study, a very high level of resistance to antibiotics among ESBL-producing Gram-negative bacteria was observed. All ESBL positive showed high resistance towards ciprofloxacin (85.1%) and cotrimoxazole (83.0%). Most ESBL producers showed higher sensitivity towards imipenem (78.7%), amikacin (73.3%), followed by piperacillin tazobactam (68.1%) (Table 4). The results of this study are similar to those of Khorvash et al. [16], which showed resistance to cefotaxime and ceftazidime and sensitivity towards carbapenems (imipenem and meropenem) (96%), and piperacillin tazobactam (84%).

8.0% were found to be MBL producers, which is higher than the earlier study conducted by Mishra et al. [6] in which MBL producers were 1.3%. In contrast with the study conducted by Shrestha et al. [25], the rate of MBL was 17.43%, which is higher. The maximum MBL activity was seen in *E. coli* (38%), *Pseudomonas* spp. (31%), followed by *K. pneumoniae* (19%) and *Proteus* spp. (12%), as in Table 3. In the study by Bora et al. [4], MBL activity was 18.9% and 21.0% for *E. coli* and *K. pneumoniae*, respectively. However, in another study conducted by Nepal et al. [17], 7% were MBL producers (22.2% *E. coli* and 55.6% *Klebsiella pneumoniae*). However, other studies conducted in different countries showed the rates of MBL production to range from 13.4 to 61.5% for *E. coli* and 33–36% for *K. pneumoniae* [18, 19].

All MBL positive organisms were found to be resistant to all primary and supplementary drugs. Most of them showed high resistance towards cefepime (80%), piperacillin tazobactam (75%), gentamycin (75%), cefoperazone sulbactam (68%) and were found to be sensitive towards amikacin (44%) and cefoperazone sulbactam (32%) (Table 5).

In this study, 3.1% were both ESBL and MBL producers. The coexistence of both ESBL and MBL production was seen in *Escherichia coli* (50%), followed by *Klebsiella pneumoniae* (16.7%), 1 *Pseudomonas* spp. (16.7%), and 1 *Proteus* vulgaris (16.7%) (Table 6). The result was statistically significant (*P* value < 0.001). This study shows a percentage less than the study done by Kaur et al. [1], where 9.2% were both ESBL and MBL producers.

The MBL producers show resistance to nearly all the generally used drugs, so it is a matter of great concern. Similar to the findings in this study, many other studies also reveal that generally the MBL positive isolates show resistance to even the carbapenems, which are used as the last resort for treatment of MDR Gram-negative bacterial infection [6]. Therefore, there is a need to institute correct antibiotics for patients infected with MBL producers and to prevent the spread of such organisms. As the choice of antibiotics for MBL producers is restricted, it is a great cause of concern.

The potential limitation of this study is that molecular, epidemiologic, and characterization of ESBL and MBL beta-lactamases were not carried out. The early detection of beta-lactamases producing isolates in a routine lab could help avoid treatment failure, as often the isolates producing this enzyme show a susceptible phenotype in routine susceptibility testing. Further strict antibiotic policies and measures could be implemented to limit the indiscriminate use of antibiotics and to minimize the emergence of multiple beta-lactamases.

## 5. Conclusion

Gram-negative bacteria are a major cause of urinary tract infections, respiratory infections, and pyogenic infections. The major causative agent in various clinical samples was *E. coli*, followed by *Klebsiella* spp. and *Pseudomonas* spp. The majority of Gram-negative isolates showed susceptibility towards nitrofurantoin, followed by gentamycin.

The prevalence of ESBL and MBL producing isolates was found to be high in Gram-negative bacteria. The increasing pattern of drug resistance was seen among ESBL and MBL producers. Most ESBL producing pathogens showed sensitivity to imipenem followed by amikacin and piperacillin tazobactam, whereas MBL producing pathogens showed sensitivity towards amikacin only.

### 5.1. Recommendations

It is essential to screen and report ESBL and MBL production along with routine susceptibility testing, which will help the clinician in prescribing proper antibiotics.

Imipenem followed by gentamycin and piperacillin tazobactam can be used as drugs for treatment of ESBL producing strains.

Amikacin can be used as drugs for treatment of MBL producing isolates.

## Figures and Tables

**Table 1:** Distribution of organisms among different clinical samples.

Organism	Sample						
	Urine	Sputum	Pus	Bile	Throat swab	Tracheal aspirate	Total
*Escherichia coli*	90	2	—	—	—	—	92
*Klebsiella* spp.	31	13	5	1	—	—	50
*Pseudomonas* spp.	12	13	1	—	—	1	27
*Citrobacter* spp.	10	2	1	—	—	—	13
*Proteus* spp.	8	—	—	—	—	—	8
*Acinetobacter lwoffi*	1	—	—	—	1	1	3
*Morganella* spp.	2	—	—	—	—	—	2
*Burkholderia cepacia*	1	—	—	—	—	—	1
*Enterobacter aerogenes*	—	—	1	—	—	—	1
Total	155	28	8	1	1	2	197

**Table 2:** ESBL production among Gram-negative isolates.

		ESBL positive	
Organism	Total	No	%
*Escherichia coli*	92	25	53
*Klebsiella pneumonia*	44	11	23
*Klebsiella oxytoca*	6	0	0
*Pseudomonas* spp	27	6	13
*Citrobacter freundi*	3	0	0
*Citrobacter koseri*	10	1	2
*Proteus vulgaris*	7	1	2
*Proteus mirabilis*	1	0	0
*Enterobacter aerogenes*	1	0	0
*Morganella morganii*	2	2	4
*Acinetobacter* spp	3	1	2
*Burkholderia cepacia*	1	0	0
Total	197	47	100

**Table 3:** MBL production among Gram-negative isolates.

		MBL positive	
Organism	Total	No	%
*Escherichia coli*	92	6	38
*Klebsiella oxytoca*	6	0	0
*Klebsiella pneumonia*	44	3	19
*Pseudomonas aeruginosa*	27	5	31
*Citrobacter freundii*	3	0	0
*Citrobacter koseri*	10	0	0
*Proteus mirabilis*	1	1	6
*Proteus vulgaris*	7	1	6
*Acinetobacter* spp	3	0	0
*Enterobacter aerogenes*	1	0	0
*Morganella morganii*	2	0	0
*Burkholderia cepacia*	1	0	0
Total	197	16	100

**Table 4:** Antibiotic resistance pattern of ESBL producers (*n* = 47).

Antibiotic used	Susceptibility pattern					
	Resistant		Intermediate		Sensitive	
	No	%	No	%	No	%
Gentamycin	20	46.5	8	18.6	16	37.2
Cotrimoxazole	39	83.0	0	0.0	8	17.0
Ciprofloxacin	40	85.1	0	0.0	7	14.9
Nitrofurantoin	15	36.6	4	9.8	20	48.8
Norfloxacin	33	78.6	1	2.4	8	19.0
Imipenem	10	21.3	0	0.0	37	78.7
Amikacin	10	22.2	2	4.4	33	73.3
Levofloxacin	28	50.0	0	0.0	28	50.0
Piperacillin tazobactam	10	21.3	5	10.6	32	68.1

**Table 5:** Antibiotic resistance pattern of MBL producers.

Antibiotic used	Susceptibility pattern					
	Resistant		Intermediate		Sensitive	
	No	%	No	%	No	%
Cefepime	4	80	0	0	1	20
Cefoperazone sulbactam	11	68	0	0	5	32
Piperacillin tazobactam	12	75	3	19	1	6
Imipenem	16	100	0	0	0	0
Ceftazidime	16	100	0	0	0	0
Gentamycin	12	75	1	6	3	19
Amikacin	9	56	0	0	7	44
Ciprofloxacin	16	100	0	0	0	0
Cotrimoxazole	16	100	0	0	0	0
Nitrofurantoin	5	56	1	6	4	25

**Table 6:** ESBL and MBL producers among the various isolates.

Organism	Total no of isolates	Both ESBL and MBL producers no. (%)	*P* value^*∗*^
*Escherichia coli*	92	3 (50)	<0.001
*Klebsiella* spp.	50	1 (16.7)	
*Pseudomonas* spp.	27	1 (16.7)	
*Citrobacter* spp.	13	0	
*Proteus* spp.	8	1 (16.7)	
*Acinetobacter* spp.	3	0	
*Morganella* spp.	2	0	
*Enterobacter* spp.	1	0	
*Burkholderia*	1	0	
Total	197	6 (3.1%)	

^*∗*^ *P* value calculated using chi-square test.

## Data Availability

The data used to support the findings of this study are available from the corresponding author upon request.

## Supplementary Materials

Supplementary Materials The Strengthening the Reporting of Observational Studies in Epidemiology (STROBE) checklist was prepared and submitted for the presentation of the research work [20] during manuscript submission for more effectiveness.
