# Supplementary material for: Detection of Beta-Lactamases (ESBL and MBL) Producing Gram-Negative Pathogens in National Public Health Laboratory of Nepal
Source: Int J Microbiol. 2022 Oct 6;2022:5474388. doi: 10.1155/2022/5474388 (PMC9560861; doi:10.1155/2022/5474388)
Supplement: Supplementary Materials — The Strengthening the Reporting of Observational Studies in Epidemiology (STROBE) checklist was prepared and submitted for the presentation of the research work [20] during manuscript submission for more effectiveness. [file 5474388.f1.doc]

STROBE checklist

|  | Item No | Recommendation |
| --- | --- | --- |
| **Title and abstract** | 1 | (*a*) Prevalence of ESBL and MBL Producing Gram Producing Gram Negative pathogens |
|  |  | (*b*) In this study, 4.6% were found to be gram negative bacteria. Among them, 50.2% isolates were found to be MDR and 23.9% were ESBL producers. The most predominant organism was *Escherichia coli*(53%), *Klebsiella pneumonia* (23%) *and*  *Pseudomonas* spp (13%). 18.2% were positive for MBL producers and 3.1% were both ESBL and MBL producers. The maximum MBL activity was seen in *E.coli* ( 38%), followed by *Pseudomonas* spp (31%) and *K. pneumoniae* (19%). |
| Introduction | | |
| Background/  Rationale | 2 | Antibiotic resistance among Gram-negative bacilli is a rapidly increasing problem due to the organisms’ ability to mutate, and to acquire and transmit plasmids and other mobile genetic elements encoding resistance gene (Kaur et al 2017). Multidrug resistance is a major health problem in Nepal that prevents the management of several infectious disease and compromises the therapy (Upadhyay A 2012).  Beta-lactamase antibiotics are the most widely prescribed antibiotics worldwide and the emergence of resistance to these agents has resulted in a major clinical crisis (Siddiqui *et al.,* 2014). There are over 340 different types of β-lactamases. These are mainly ESBLs, Amp C and carbapenemases. ESBLs are still considered as a threat since they are coded by plasmid and can be easily transmitted between species Carbapenems are used as drug of choice to treat infections caused by ESBL producing bacteria. However over the past few years, carbapenem resistance due to metallo-beta-lactamases (MBLs) production has been increasingly reported among clinical isolates from all around the world [Bora A 2014 ]. MBLs have been globally isolated from various bacteria and more than 80 types of MBLs have been identified worldwide, with over 75% occurring as plasmid encoded enzymes (Bonnet 2004).  The aim of the current study is to determine the prevalence of ESBL and MBL producers among gram negative clinical isolates. Early detection of ESBL and MBL producing organisms is crucial to establish appropriate antimicrobial therapy and to prevent their inter hospital dissemination.  Nepal is one of the developing country in South East Asia where the antibiotics can be obtained and used without medical authorization or supervision. Here, financial problem is the main factor that adversely affects the regular health checkups and people usually seek for medical services only when the symptoms of the disease start to become more evident. This situation has led to inappropriate usage of antibiotics with patients taking the drugs for insufficient length of time or at sub optimal dosages, which may result in antimicrobial resistance. So, this study will also help clinicians in order to facilitate the empirical treatment of patients and management of patients with symptoms . |
| Objectives | 3 | To isolate and identify the pathogenic gram negative bacterial isolates.  To assess the antibiotic susceptibility pattern of the isolates.  To screen and detect the Extended Spectrum Beta Lactamase (ESBL) producers among the isolates.  To screen and detect Metallo-Beta Lactamase (MBL) producers among the isolates. |
| Methods | | |
| Study design | 4 | Descriptive study |
| Setting | 5 | This was cross-sectional study conducted at Bacteriology Department of National Public Health Laboratory (NPHL), Teku, Nepalduring the period of October 2017 to March 2018. |
| Participants | 6 | (*a*) all patient suspected of microbial infection  Give the eligibility criteria, and the sources and methods of selection of participants |
| Variables | 7 | Dependent variables : different clinical isolates like *Escherichia coli, Klebsiella ,Citrobacter, Pseudomonas, Proteus vulgaris* etc, ESBL, MBL, AST  Independent variables: age , sex, education |
| Data sources/ measurement | 8* |  |
| Bias | 9 | No potential bias |
| Study size | 10 | about 400 n formula is at 95% Cl Sample size=z2 × std dev (1- Std dev) / d2 Where z score= 1.96 Std dev= 0.5 d= precision |
| Quantitative variables | 11 |  |
| Statistical methods | 12 | (*a*) Describe all statistical methods, including those used to control for confounding |
|  |  | (*b*) Describe any methods used to examine subgroups and interactions |
|  |  | (*c*) no missing data |
|  |  | (*d*) |
|  |  | (*e*) Describe any sensitivity analyses |
| Results | | |
| Participants | 13* | (a) Report numbers of individuals at each stage of study—eg numbers potentially eligible, examined for eligibility, confirmed eligible, included in the study, completing follow-up, and analysed  Sample was 400 but the sample flow is very low all samples during the peiod of four months were included in the study. Total 4266 clinical specimens (urine, body fluid, pus, bile, tracheal aspirate, throat swab and sputum) were received and processed for routine culture and susceptibility testing. |
|  |  | (b) Give reasons for non-participation at each stage  NPHL is not hospital and it hasnot its own patient . it is  the  government reference laboratory which is  controlling body for other  laboratories ( periphery and private ). As patient  flow is less, all ages of patient suspected of microbial infection were included for the study. |
|  |  | (c) Consider use of a flow diagram |
| Descriptive data | 14* | 1. Give characteristics of study participants (eg demographic, clinical, social) and information on exposures and potential confounders   Samples from persons suspected of microbial infection were included in this study and there was no potential harm on them as patient giving only their samples |
|  |  | (b) Indicate number of participants with missing data for each variable of interest  No missing data |
| Outcome data | 15* | Report numbers of outcome events or summary measures  197 gram negative bacteria were isolated from the culture and further processed for MDR, AST ESBL and MBL detection. |
| Main results | 16 | (*a*) Give unadjusted estimates and, if applicable, confounder-adjusted estimates and their precision (eg, 95% confidence interval). Make clear which confounders were adjusted for and why they were included |
|  |  | Report category boundaries when continuous variables were categorized |
|  |  | (*c*) If relevant, consider translating estimates of relative risk into absolute risk for a meaningful time period |
| Other analyses | 17 | Report other analyses done—eg analyses of subgroups and interactions, and sensitivity analyses  MDR Detection of esbl mbl and AST were performed |
| Discussion | | |
| Key results | 18 | In this study, 4.6% were culture positive . few other studies carried out by Nepal et al (2017), Mishra et al (2012) and Thapa et al (2017) showed higher percentage of growth positivity of 29.9%, 17.1%, 44.4% and 13.9% respectively. Among the total 197 bacterial isolates, 9 different bacterial species were isolated, , *E.coli* 92(46.7%) was found to be the most predominant organism. Higher prevalence of *E. coli* seen in this study which also resembled the study done by various other authors viz: Khadka et al 2010(50%), Mishra et al 2008(69.6%), Chander and Shrestha(63.3%) , Raut et al 2015(83.8%), Nepal et al 2017(51.5%) and Thapa et al 2017 (54.9%) in Nepal. In this study, the antibiotic sensitivity pattern of gram negative isolates showed increase resistance to ampicillin (91.8%) which is in harmony with the resistance pattern of pathogens in study of Shrestha et al (2012) Kattel et al (2012), Raut et al (2015), Nepal et al (2017). Nitrofurantoin (55.5%) was found to be the most effective antibiotic against bacteria followed by Gentamycin (53.0%) and Cefotaxime (50.8%) respectively  47 (23.9%) were ESBL producers The result of prevalence of ESBL producing Gram negative bacteria were higher than the study carried out by Pokhrel et al (2006) in which 16.0 % isolates were found to be ESBL producers and also to the study done by Ahmed et al (2014) in which 18.95% were ESBL producers. In this study *E coli (*53%) was the major ESBL producer followed by *K pneumoniae*(23%), *Pseudomonas* spp(6%), *Morganella morganii*(4%*) .*  Of 197 bacterial isolates, 28 were found to be MBL screening test positive and were subjected to MBL confirmatory test. 16/197( 8.0 %) were found to be MBL producers which is higher than the earlier study conducted by Mishra et al (2012) in which MBL producers were 1.3%. In contrast with the study conducted by Shrestha et al the rate of MBL was 17.43% which is higher. |
| Limitations | 19 | Discuss limitations of the study, taking into account sources of potential bias or imprecision. Discuss both direction and magnitude of any potential bias  Limitation of this study is the molecular confirmation of bacteria, esbl as well as MBL . |
| Interpretation | 20 | Give a cautious overall interpretation of results considering objectives, limitations, multiplicity of analyses, results from similar studies, and other relevant evidence  The growth of gram negative bacteria is 4.6% . *E.coli* 92(46.7%) was found to be the most predominant organism followed by *Klebsiella* spp 50 (25.4%), *Pseudomonas* spp 2(13.7%)*, Citrobacter* spp 13 (6.6%), *Proteus* spp 8(4.06%), *Acinetobacte*r spp 3(1.5%), *Morganella* spp 2(1.01%), *Enterobacter* spp 1(0.5%), *Burkholderia* spp 1(0.5%) respectively. Their antibiotic sensitivity pattern showed that they were resistant to Ampicillin (91.8%), Ciprofloxacin (57.7%), Norfloxacin (57.3%), Cotrimoxazole (50.0%), and Cefotaxime (49.2%) respectively. Among them, 23.9% were ESBL producers 8.0 % were MBL producers All ESBL positive showed high resistance towards Ampicillin (100%) followed by Cefotaxime (100%), Ceftazidime (91.5%), Ciprofloxacin (85.1%) and Cotrimoxazole (83.0%). Most ESBL producers showed higher sensitivity towards Imipenem (78.7%), Amikacin (73.3%) followed by Piperacillin+tazobactam (68.1%). MBL producers were resistant to all primary and supplementary drugs. Most of them showed high resistance towards Cefepime (80%), Piperacillin + Tazobactam (75%), Gentamycin (75%), Cefoperazone + Sulbactam (68%) and were found to be sensitive towards Amikacin (44%) and Cefeperozone+ sulbactam(32%).  Limitation of this study is the molecular confirmation of bacteria, esbl as well as MBL . |
| Generalisability | 21 | Different ATCC Strains were used for the quality control of stain, media preparation, isolation and identification, Antibiotic susceptibility pattern. The laboratory equipment were regularly monitored for their efficiency. The temperature of refrigerator and incubator were monitored and documented every day. |
| Other information | | |
| Funding | 22 | No funding |

*Give information separately for exposed and unexposed groups.

**Note:** An Explanation and Elaboration article discusses each checklist item and gives methodological background and published examples of transparent reporting. The STROBE checklist is best used in conjunction with this article (freely available on the Web sites of PLoS Medicine at http://www.plosmedicine.org/, Annals of Internal Medicine at http://www.annals.org/, and Epidemiology at http://www.epidem.com/). Information on the STROBE Initiative is available at www.strobe-statement.org.
